# Methylglyoxal-induced glycation changes adipose tissue vascular architecture, flow and expansion, leading to insulin resistance

**DOI:** 10.1038/s41598-017-01730-3

**Published:** 2017-05-10

**Authors:** Tiago Rodrigues, Paulo Matafome, José Sereno, José Almeida, João Castelhano, Luís Gamas, Christian Neves, Sónia Gonçalves, Catarina Carvalho, Amina Arslanagic, Elinor Wilcken, Rita Fonseca, Ilda Simões, Silvia Vilares Conde, Miguel Castelo-Branco, Raquel Seiça

**Affiliations:** 1 Laboratory of Physiology, CNC.IBILI and Faculty of Medicine, University of Coimbra, Coimbra, Portugal; 2 Instituto Politécnico de Coimbra, Coimbra Health School (ESTeSC), Department of Complementary Sciences, Coimbra, Portugal; 3 Institute of Nuclear Sciences Applied to Health (CIBIT-ICNAS), University of Coimbra, Coimbra, Portugal; 4 Serviço de Anatomia Patológica, University Hospital Center of Coimbra, Coimbra, Portugal; 5 CEDOC, NOVA Medical School - Faculty of Medical Sciences, New University of Lisbon, Lisbon, Portugal; 6 Laboratory of Visual Neuroscience, CNC.IBILI and Faculty of Medicine, University of Coimbra, Coimbra, Portugal

## Abstract

Microvascular dysfunction has been suggested to trigger adipose tissue dysfunction in obesity. This study investigates the hypothesis that glycation impairs microvascular architecture and expandability with an impact on insulin signalling. Animal models supplemented with methylglyoxal (MG), maintained with a high-fat diet (HFD) or both (HFDMG) were studied for periepididymal adipose (pEAT) tissue hypoxia and local and systemic insulin resistance. Dynamic contrast-enhanced magnetic resonance imaging (DCE-MRI) was used to quantify blood flow *in vivo*, showing MG-induced reduction of pEAT blood flow. Increased adipocyte size and leptin secretion were observed only in rats feeding the high-fat diet, without the development of hypoxia. In turn, hypoxia was only observed when MG was combined (HFDMG group), being associated with impaired activation of the insulin receptor (Tyr1163), glucose intolerance and systemic and muscle insulin resistance. Accordingly, the adipose tissue angiogenic assay has shown decreased capillarization after dose-dependent MG exposure and glyoxalase-1 inhibition. Thus, glycation impairs adipose tissue capillarization and blood flow, hampering its expandability during a high-fat diet challenge and leading to hypoxia and insulin resistance. Such events have systemic repercussions in glucose metabolism and may lead to the onset of unhealthy obesity and progression to type 2 diabetes.

## Introduction

Recent studies suggested different metabolic outcomes of metabolically healthy obesity (MHO) and metabolically unhealthy obesity (MUO), the latter being more likely associated with early development and progression of type 2 diabetes^1–3^. Adipose tissue dysfunction seems to be pivotal in this different outcome, given its contribution to insulin resistance and glucose and lipid dysmetabolism and the multilevel impact of adipokines^4–9^. Studies using genetic (*ob*/*ob* or *db*/*db*) and diet-induced models of obesity have shown that lipotoxicity-triggered inflammation and hypoxia govern adipose tissue dysfunction, causing lipolysis, ectopic FFA deposition and insulin resistance^4, 6, 10–12^. Models of high-fat (HF) diet-induced obesity are considered more physiological. However, hypercaloric diets are usually composed by a mixture of lipids and sugars, which may cause adipose tissue dysfunction without isolating the mechanisms involved.

Hypoxia was suggested to primarily derive from adipocyte hypertrophy, which would limit oxygen diffusion^12, 13^. However, recent evidences support the idea that hypoxia is not a primary event and may result from other factors than simple adipocyte growth^14^. Limited capillarization was recently shown in adipose tissue explants from obese donors, potentially causing hypoxia. However, reduced adipose tissue blood flow was observed to be independent of obesity *per se*, and shown to be more associated with insulin resistance^15–18^. Moreover, Goossens *et al*., recently demonstrated hyperoxia in the adipose tissue of obese patients, due to decreased metabolic rate and possibly mitochondrial dysfunction^14^. Thus, although these aspects remain to be addressed at the mechanistic level, such evidences suggest the modulation of angiogenesis and blood flow as potential targets in improving insulin sensitivity.

Methylglyoxal (MG) is a strong precursor of advanced glycation end products (AGE), which forms from glucose and thus its levels are elevated in prediabetic and diabetic patients and in sugar-rich foods^19–23^. Previous studies demonstrated MG-dependent inhibition of the insulin receptor pathway in 3T3-L1 adipocytes^24, 25^. The same was observed in animal models using fructose supplementation and different methods of intraperitoneal, intravenous and continuous subcutaneous MG administration^26–28^. In a study of Hofmann *et al*., the consumption of AGE- and MG-enriched diets caused glucose and insulin intolerance, but only in diabetic *db*/*db* mice, with much smaller effects on normal mice^29^. However, this was only observed with supraphysiological MG doses. Thus, the major limitation of the existing studies is that, to our knowledge, none of them assessed the effects of MG accumulation in comparison with similar endogenous levels observed in a diabetic model. Recently, we demonstrated that dietary MG supplementation increases AGE deposition in periepididymal adipose tissue (pEAT), causing structural and functional alterations^30^. Moreover, MG impairs metabolic adaptations after a surgery-induced decrease of blood supply and when reduced by pyridoxamine the microvascular lesions were improved^31, 32^. However, the effects of AGE deposition on the vascular adaptation in the course of adipose tissue expansion during obesity were not previously addressed. We hypothesized that progressive AGE accumulation in adipose tissue may hamper capillarization, blood flow and expandability, which could contribute to insulin resistance and thus further enhance AGE deposition in a positive feedback cycle. Understanding the mechanisms of microvascular dysfunction may definitively contribute to design strategies to improve adipose tissue function and thus prevent unhealthy obesity and type 2 diabetes development and progression.

## Results

### Glycation increases glycoconjugates and fibrosis in adipose tissue

**Figure 1:**
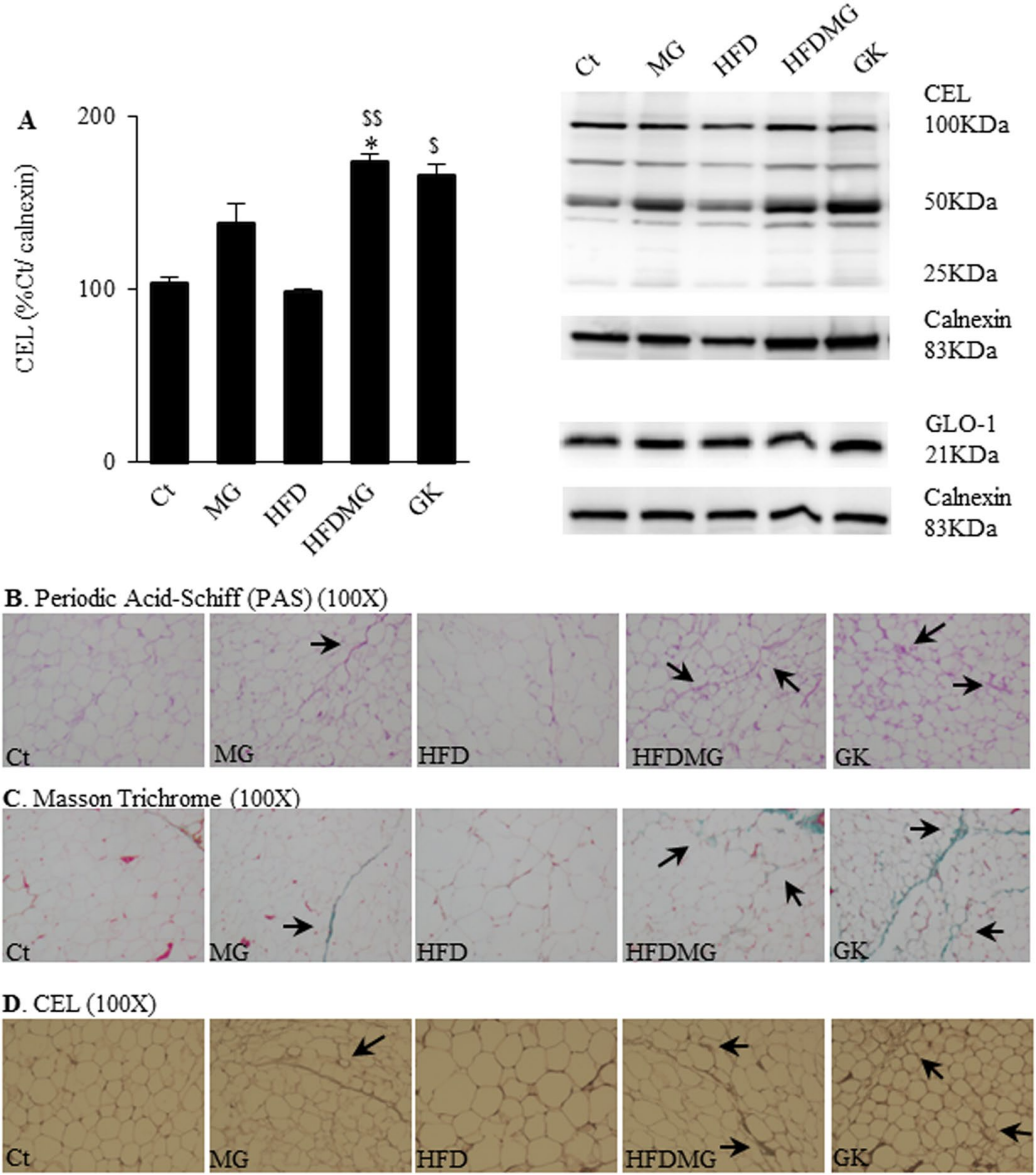
Increased pEAT levels of CEL in HFDMG and GK rats (**A**), calculated as percentage of Ct, while no differences were observed for GLO-1; representative WB are shown. Glycation increases glycoconjugates, fibrosis and CEL staining in pEAT. Histological analysis shows PAS (100X) (**B**), Masson Trichrome (100X) (**C**) and CEL staining (100X) (**D**). Ct - Wistar 12 m; MG - Wistar + MG supplementation; HFD - HF diet-fed Wistar; HFDMG - HF diet-fed Wistar + MG supplementation; GK - Goto-Kakizaki 12 m. Bars represent means ± SEM, n = 6–8. * vs Ct; ^$^ vs HFD. 1 symbol p < 0.05; 2 symbols p < 0.01.

N^e^(carboxyethyl)lysine (CEL) is an AGE specifically derived from the MG reaction with lysine residues, which may accumulate in adipose tissue from local formation or dietary absorption of MG-lysine adducts. CEL levels were significantly superior in HFDMG and GK groups (Fig. 1A). No significant alterations were found for glyoxalase-1 (GLO-I) levels. Increased PAS (glycoconjugates), Masson Trichrome (fibrosis) and CEL staining were observed in MG-treated groups (MG and HFDMG) and in the non-obese type 2 diabetic GK rats (Fig. 1B,C,D).

### Glycation impairs adipose tissue blood flow and causes hypoxia in diet-induced obese rats

**Figure 2:**
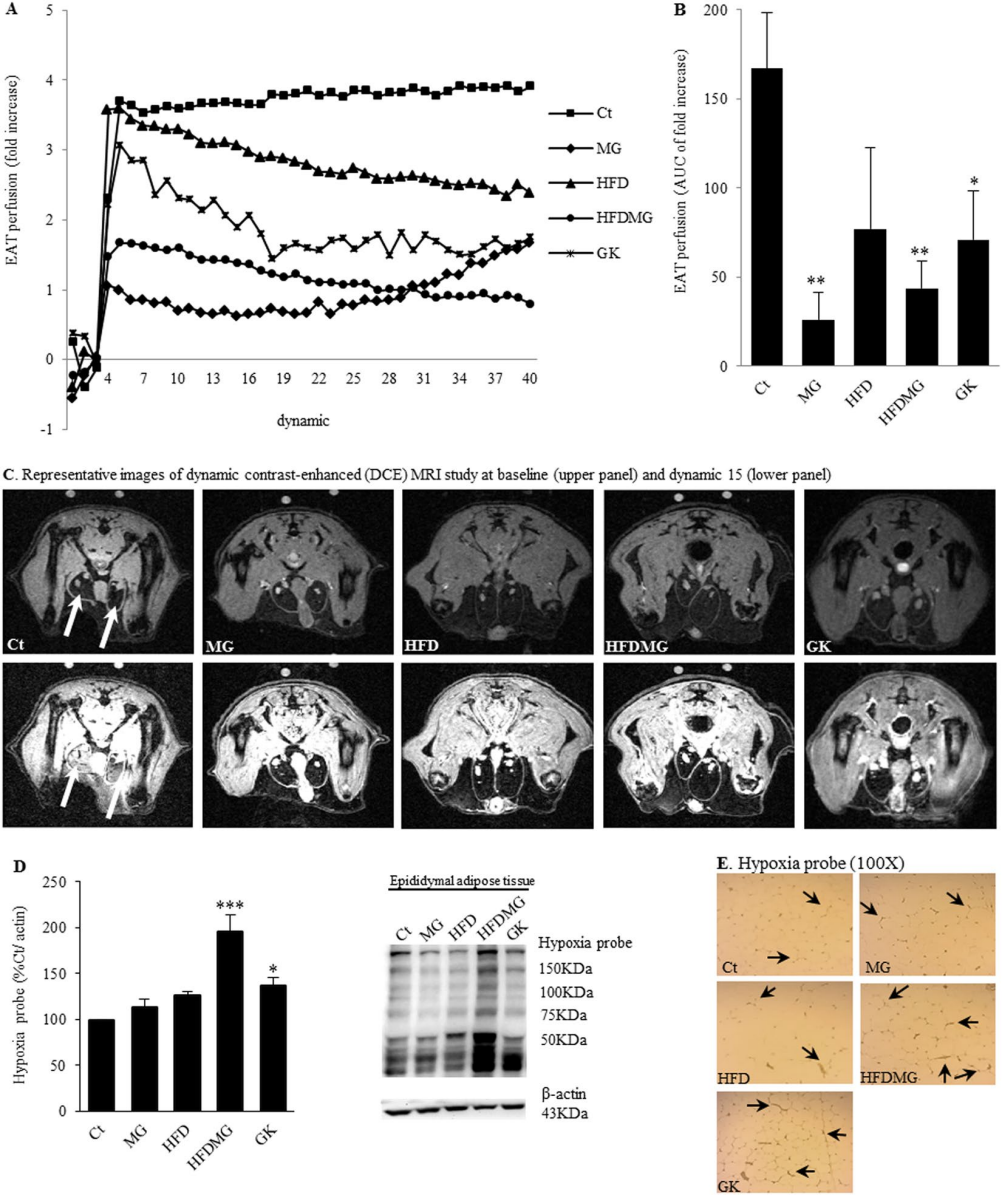
Glycation impairs pEAT blood flow and causes hypoxia in adipose tissue. Blood flow was evaluated through the area under the curve (**B**) of a dynamic contrast-enhanced (DCE) MRI study (**A**). Representative images at baseline and dynamic 15 (**C**). Hypoxia was assessed using an antibody against pimonidazole adducts by WB quantification (**D**) and IHC (**E**). Ct - Wistar 12 m; MG - Wistar + MG supplementation; HFD - HF diet-fed Wistar; HFDMG - HF diet-fed Wistar + MG supplementation; GK - Goto-Kakizaki 12 m. Bars represent means ± SEM, n = 6–8. * vs Ct. 1 symbol p < 0.05; 2 symbols p < 0.01; 3 symbols p < 0.001.

For the first time, we were able to develop a procedure to evaluate adipose tissue blood flow *in vivo* using DCE-MRI (Fig. 2). The accumulation curve of the contrast product was evaluated during 34 minutes. The fold increase in relation to the basal signal was calculated at each dynamic scan and the area under the curve (AUC) was determined. Enhancement curves of representative animals of each group (Fig. 2A) show rapid contrast product accumulation in pEAT, which is higher in control rats and reduced in groups submitted to MG supplementation (MG, HFDMG). Accordingly, the AUC was significantly reduced in these groups and in GK rats, showing decreased pEAT blood flow (Fig. 2B). Hypoxia was quantified through the accumulation of pimonidazole adducts and no significant differences were observed in HFD and MG groups (Fig. 2D). On the other hand, the HFDMG group showed increased pimonidazole accumulation (p < 0.001 vs Ct), which has also been demonstrated in histological analysis (Fig. 2D,E). Notably, hypoxia was also observed in non-obese diabetic GK rats.

### Glycation hampers adipose tissue pathways of adaptation to hypoxia, angiogenesis (capillarization) and expandability

**Figure 3:**
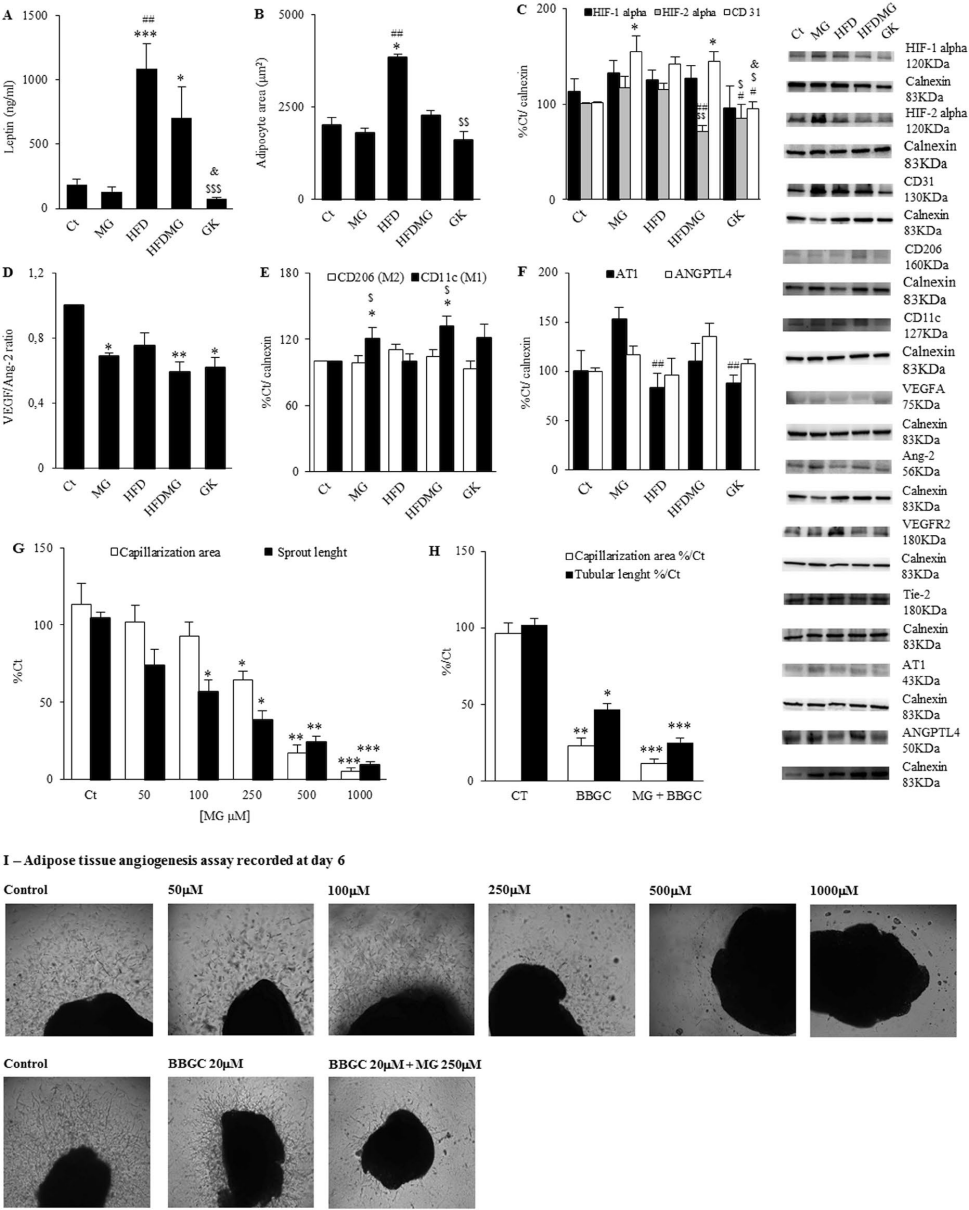
Glycation hampers adipose tissue pathways of adaptation to hypoxia, angiogenesis and expandability ratio. Serum leptin levels were assessed by ELISA (**A**). Adipocyte area was evaluated through the number of adipocytes counted in each field (10 fields/rat) (**B**). The levels of HIF-1alpha, HIF-2alpha, CD31 (**C**), VEGF, Ang-2 (**D**), CD11c, CD206 (**E**) ANGPTL4 and AT1 (**F**) were determined by WB. The VEGF/Ang-2 ratio was calculated. The adipose tissue angiogenesis assay was used to evaluate the effects of glycation in adipose tissue angiogenesis. MG concentrations higher than 100 μM decreased vascularization area, while lower concentrations decreased sprout length (**G**). Glyoxalase-1 inhibitor BBGC reduced vascularization area and sprout length, an effect which was further increased in combination with MG exposure (**H**). Representative images of all conditions are shown in (**I**). Ct - Wistar 12 m; MG - Wistar + MG supplementation; HFD - HF diet-fed Wistar; HFDMG - HF diet-fed Wistar + MG supplementation; GK - Goto-Kakizaki 12 m. Bars represent means ± SEM, n = 6–8. * vs Ct; ^#^ vs MG; ^$^ vs HFD; ^&^ vs HFDMG; 1 symbol p < 0.05; 2 symbols p < 0.01; 3 symbols p < 0.001.

pEAT expansion was assessed through the fat pad weight, adipocyte area and indirectly by circulating leptin. HFD rats had increased body and pEAT weight (Table 1). The HFDMG group showed a smaller increase of pEAT weight and no differences in body weight, although they had eaten similar amounts of food as compared to the HFD group (Table 1). GK rats had lower body weight than Wistar rats, without major differences in pEAT weight (Table 1). HF diet-induced adipose tissue expansion (HFD group) caused increased adipocyte area and circulating leptin levels (p < 0.001 vs Ct; p < 0.01 vs MG), which were not observed in the HFDMG group (leptin: p < 0.05 vs Ct) (Fig. 3A,B).

**Table 1:** Food intake, body and periepididymal adipose tissue weight, fasting glycemia, HbA1c and serum triglycerides.

Group	Ct	MG	HFD	HFDMG	GK
Food (g/rat/day)	22.9 ± 0.7	24.3 ± 1.6	15.1 ± 1.1**^,##^	14.4 ± 0.7**^,##^	25.7 ± 0.6^$$$,&&&^
Body weight (g)	508.8 ± 11.4	508.9 ± 18.4	652.7 ± 35.8**^,##^	571.6 ± 27.3	398.7 ± 7.7*^,#,$$$,&&&^
pEAT weight (g)	5.5 ± 0.4	5.9 ± 1.1	16.8 ± 2.5**^,#^	14.2 ± 1.4*	2.8 ± 0.3^###^^,^^&&&^
Fasting glycemia (mg/dl)	68.5 ± 2.0	70.6 ± 1.4	70.9 ± 2.0	71.1 ± 1.6	91.7 ± 3.0***^,#,$,&^
HbA1c (%) (mmol/mol)	3.2 ± 0.1 (11.8 ± 1.5)	3.3 ± 0.1 (13 ± 2.1)	3.3 ± 0.1 (12.8 ± 0.9)	3.5 ± 0.1 (15.4 ± 1.9)	5.6 ± 0.4 (36 ± 6.8)^***,###,$$$,&&&^
Triglycerides (mg/dl)	77.2 ± 6.4	69.3 ± 10.7	77.8 ± 5.4	62.3 ± 3.2	160.1 ± 23.1***^,###,$$$,&&&^

Ct - Wistar 12 m; MG - Wistar + MG supplementation; HFD - HF diet-fed Wistar; HFDMG - HF diet-fed Wistar + MG supplementation; GK - Goto-Kakizaki 12 m. Average ± SEM.* vs Ct; ^#^ vs MG; ^$^ vs HFD; ^&^ vs HFDMG. 1 symbol p < 0.05; 2 symbols p < 0.01; 3 symbols p < 0.001.

Adequate angiogenesis is determinant for adipose tissue expandability. When hypoxia regions are generated by physiological tissue expansion, hypoxia-inducible factors (HIFs) are activated as a mechanism to increase angiogenesis. Despite the fact that no changes were observed in HIF-1alpha, decreased HIF-2alpha expression in HFDMG and GK groups were observed when comparing with MG and HFD groups (Fig. 3C). Decreased HIF-2alpha expression in adipose tissue has been associated with activation of the macrophage M1 phenotype. Accordingly, HFDMG rats had increased M1 levels (Fig. 3E). No differences were observed for the M2 phenotype. Besides increased number of M1 macrophages, dysregulation of HIFs expression may hamper angiogenesis as well. Our group showed aberrant capillary formation in RPE cells and pEAT after MG-induced HIF-1alpha degradation and imbalance of VEGF/Ang-2 ratio^30, 33^. A similar imbalance of VEGF/Ang-2 ratio was here observed in the MG-supplemented groups and GK rats (Fig. 3D). This was coincident with increased levels of the endothelial cell marker CD31 in MG and HFDMG groups, what may denote a compensatory endothelial cell proliferation and formation of aberrant capillaries^33^ (Fig. 3C). Such results were corroborated by the adipose tissue angiogenesis assay. Incubation of adipose tissue explants with growing concentrations of MG showed progressive inhibition of endothelial cell migration in the collagen matrix only for concentrations higher than 100 μM (Fig. 3G). However, concentrations between 50 μM and 100 μM MG caused a significant decrease of sprout length, showing that glycation-induced vessel destabilization precedes inhibition of cell proliferation and migration in the adipose tissue (Fig. 3G). Selective inhibition of GLO-1 also caused a significant reduction of vascularization area and sprout length, an effect which was further increased when it was combined with 250 μM MG (Fig. 3H). Besides hampering angiogenesis, MG supplementation also increased angiotensin II receptor (AT1) expression, given that the HF diet decreased AT1 levels in adipose tissue but such decrease was not observed in HF diet-fat rats supplemented with MG (Fig. 3F). Such effects may increase angiotensin signalling decreasing blood flow.

### Glycation impairs adipose tissue insulin signalling in high-fat diet-fed rats

**Figure 4:**
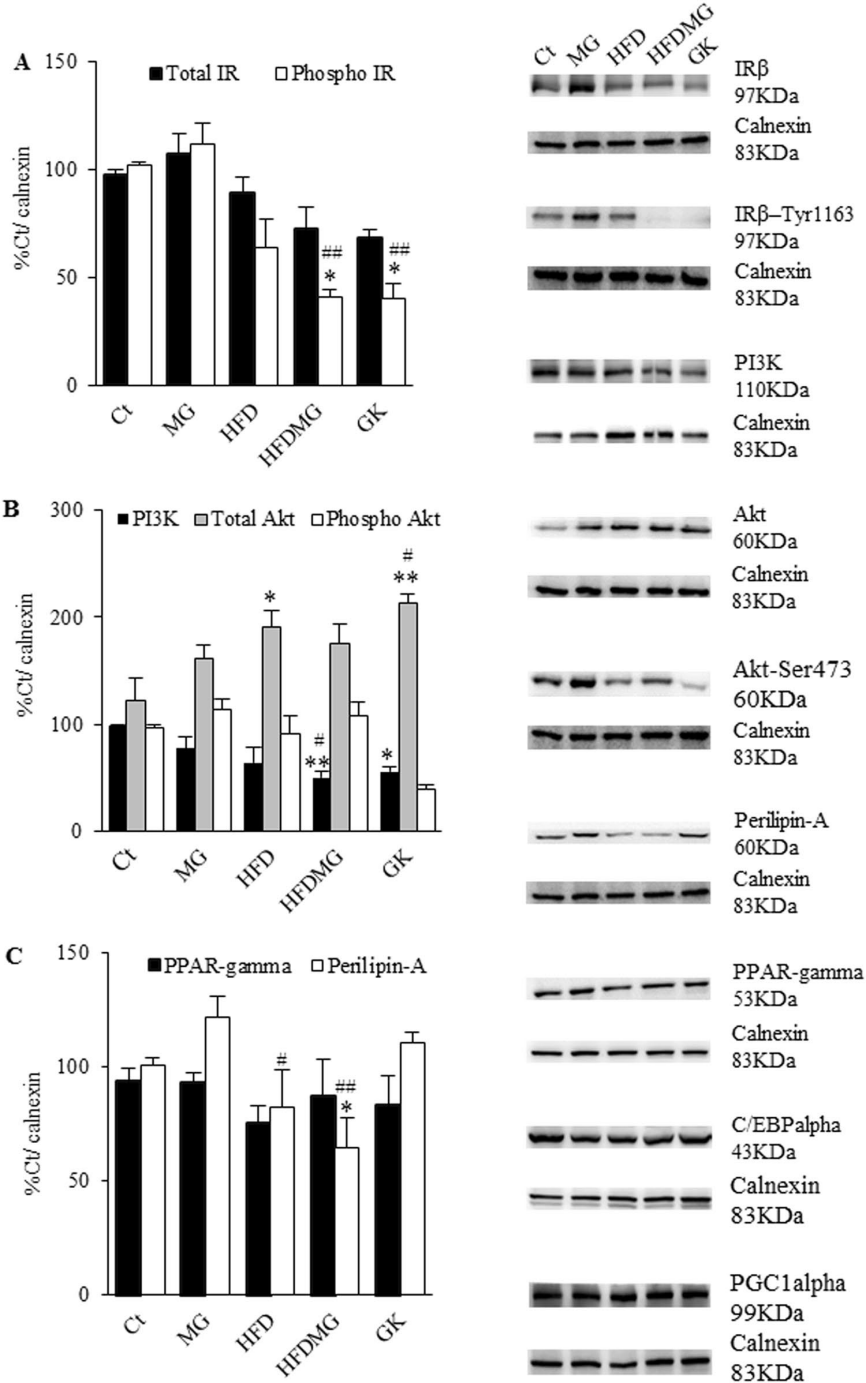
Glycation impairs pEAT insulin signalling in high-fat diet-fed rats. Insulin signalling in pEAT was evaluated through WB quantification of activated and total forms of IR (**A**) and Akt (**B**). pEAT metabolism was assessed by WB quantification of PPARgamma and Perilipin-A levels (**C**). Ct - Wistar 12 m; MG - Wistar + MG supplementation; HFD - HF diet-fed Wistar; HFDMG - HF diet-fed Wistar + MG supplementation; GK - Goto-Kakizaki 12 m.Bars represent means ± SEM, n = 6–8.* vs Ct; ^#^ vs MG; 1 symbol p < 0.05; 2 symbols p < 0.01.

Methylglyoxal supplementation and HF diet separately had no major effects in the insulin receptor, Akt, PPARgamma (regulator of lipid storage) and Perilipin-A (regulator of lipolysis) levels. However, HF diet with MG supplementation induced a significant decrease of activated insulin receptor form, similarly to non-obese type 2 diabetic rats (p < 0.05 vs Ct and p < 0.01 vs MG) (Fig. 4A). No major differences were however observed in phosphorylated Akt, PGC1alpha and the differentiation factors PPAR-gamma and C/EBPalpha (Fig. 4B,C). Perilipin-A degradation is strongly inhibited by insulin and, accordingly, its levels were significantly reduced in the HFDMG group suggesting insulin resistance (Fig. 4C). Thus, impairment of pEAT expandability induced by glycation results in impaired insulin signalling and lipid storage.

### Glycation causes systemic dysmetabolism and impairs skeletal muscle insulin signalling in high-fat diet-fed rats

**Figure 5:**
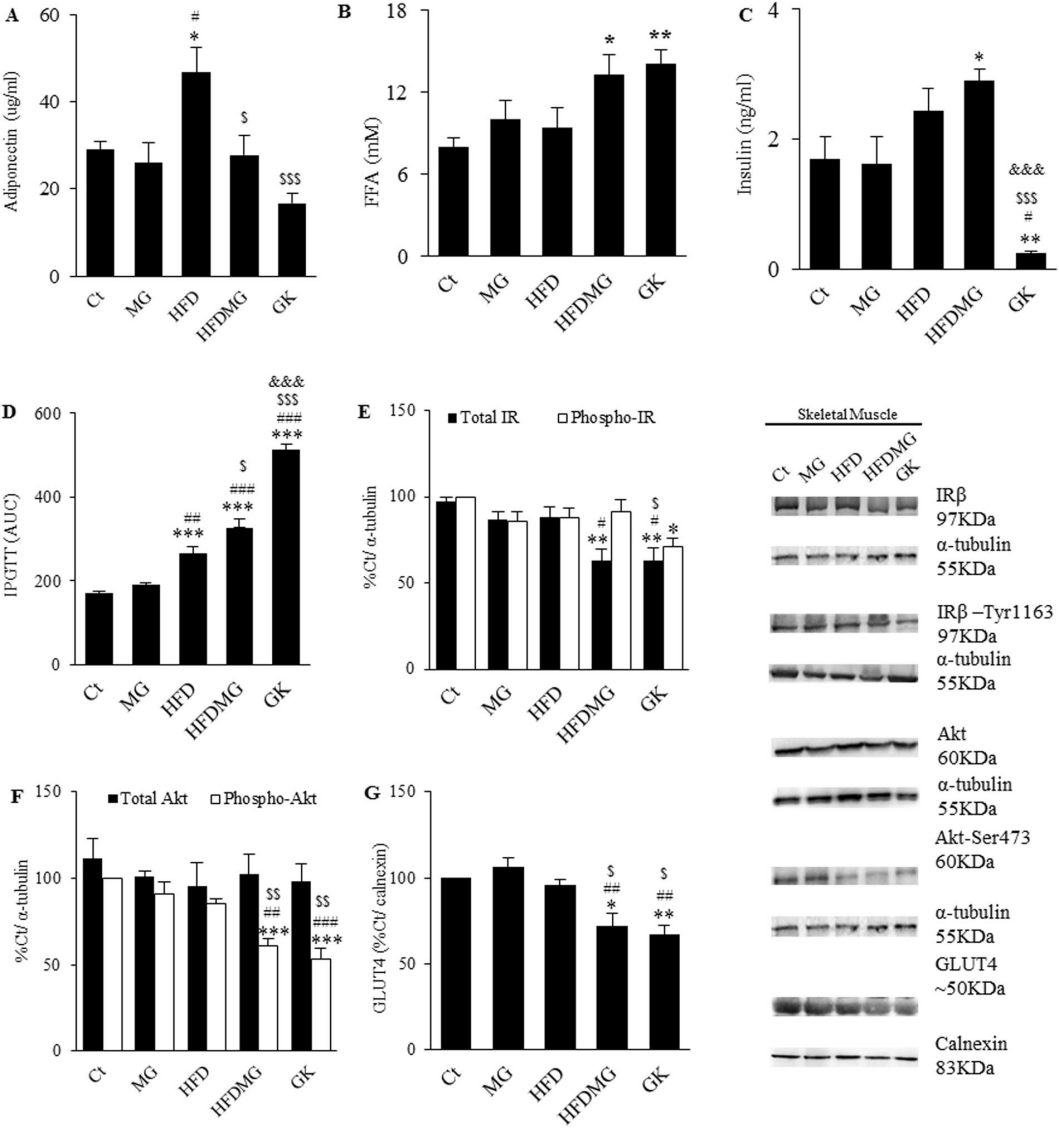
Glycation causes systemic dysmetabolism and impairs skeletal muscle insulin signaling in high-fat diet-fed rats. Circulating levels of adiponectin (**A**), FFAs (**B**) and insulin (**C**) were quantified by ELISA. Glucose intolerance was evaluated through the area under the curve of an IPGTT (**D**). Skeletal muscle insulin signalling was evaluated through WB quantification of activated and total forms of IR (**E**) and Akt (**F**) as well as GLUT4 transporter (**G**). Ct - Wistar 12 m; MG - Wistar + MG supplementation; HFD - HF diet-fed Wistar; HFDMG - HF diet-fed Wistar + MG supplementation; GK - Goto-Kakizaki 12 m. Bars represent means ± SEM, n = 6–8. * vs Ct; ^#^ vs MG; ^$^ vs HFD; & vs HFDMG; 1 symbol p < 0.05; 2 symbols p < 0.01; 3 symbols p < 0.001.

Methylglyoxal and HF diet effects on systemic metabolism were assessed through the evaluation of glycemia (fasting and IPGTT), HbA1c, FFA, triglycerides, insulin and adiponectin. MG supplementation alone had no effect on such systemic parameters (Fig. 5A–D; Table 1). Despite increased adiponectinemia, HFD rats developed glucose intolerance, with higher AUC during the IPGTT (p < 0.001 vs Ct and p < 0.01 vs MG), but no significant differences were observed for HbA1c, fasting glycemia, insulinemia and FFA levels (Fig. 5A–D; Table 1). In turn, HFDMG rats developed higher fasting FFA levels (p < 0.05 vs Ct), insulinemia (p < 0.05 vs Ct) and glucose intolerance (AUC) (p < 0.05 vs HFD; p < 0.001 vs Ct and MG) (Fig. 5B–D). Moreover, MG-induced glycation inhibited the increase of serum adiponectin levels observed in the HFD group (p < 0.05 vs HFD) (Fig. 5A). Such features were similar to the type 2 diabetic rats, which develop glucose intolerance, hypoadiponectinemia and increased FFA levels, as well as hypoinsulinemia, due to age-dependent impaired β-cell function (Fig. 5A–D). Altogether, such results show that glycation in HF diet fed-rats results in systemic insulin resistance and impaired glucose tolerance.

As the adipose tissue, the skeletal muscle is a main target of insulin. MG supplementation or the HF diet separately had no effects in insulin signalling of skeletal muscle (Fig. 5E–G). However, HF diet fed-rats submitted to MG-supplementation demonstrated a significant decrease in the levels of the insulin receptor (p < 0.05 vs MG; p < 0.01 vs Ct), Akt active form (p < 0.01 vs MG and HFD; p < 0.001 vs Ct) and GLUT4 (p < 0.05 vs Ct and HFD; p < 0.01 vs MG) (Fig. 5E–G). Such effects were very similar to GK rats, which also showed decreased phopho-IR, total-IR, phospho-Akt and GLUT4 (Fig. 5E–G). Such results show that glycation also impairs skeletal muscle insulin signalling, contributing to systemic insulin resistance and glucose intolerance.

## Discussion

In this study we investigated a new mechanism for adipose tissue dysfunction in obesity and type 2 diabetes. We demonstrate that glycation in pEAT has adverse vascular effects, impairing blood flow, hypoxia-response mechanisms and expandability which is tightly associated with local and systemic insulin resistance and glucose dysmetabolism. Adipose tissue dysfunction has been suggested to be caused by limited adipose tissue angiogenesis and expansion potential^17, 34^. This may be of particular relevance due to different metabolic outcomes of metabolically healthy (MHO) and unhealthy obesity (MUO). The latter is characterized by the earlier progression to insulin resistance and glucose dysmetabolism^1, 2, 35^.

Previously, we have shown that MG supplementation to Wistar rats impairs angiogenic markers and blood flow in adipose tissue, causing hypoxia, macrophage recruitment, hypoadiponectinemia and increased plasma FFA’s, but neither insulin resistance nor glucose dysmetabolism^30^. Such effects are reverted by pyridoxamine treatment, a dicarbonyl scavenger drug^31^. We have also demonstrated that glycation impairs adipocyte ability to adapt to hypoxia in a model of surgery-induced reduction of blood supply to the left pEAT, causing insulin resistance and adipocyte death^32^. Based on such observations, we hypothesized that adipose tissue glycation may induce microvascular lesions that hamper blood flow and expandability during a diet-induced expansion and lead to adipose tissue dysfunction and insulin resistance in obesity. This would therefore provide a strong mechanistic framework to the MUO phenotype. To test this conceptual model, we developed an animal model with HF diet-induced adipose tissue expansion and MG supplementation. We used a diet specifically enriched in triglycerides in order to induce physiological adipose tissue expansion and better isolate the variables of interest, while controlling for potential confounds. The effects of MG were compared with the endogenous glycation observed in diabetic GK rats.

MG supplementation in the diet increases the formation of stable MG adducts that are partially absorbed to the bloodstream, accumulate in different tissues and cause diabetes-like microvascular lesions^36, 37^. Our group demonstrated that our protocol results in MG levels (after derivatization) in plasma and adipose tissue that are similar to those of diabetic rats and here we demonstrate that MG supplementation results in CEL levels in adipose tissue similar to diabetic rats^30^.

In order to evaluate adipose tissue blood flow, we have developed, validated and applied a new *in vivo* quantitative DCE-MRI technique to assess pEAT blood flow. In the past, we evaluated pEAT blood flow through the accumulation of the fluorescent dye Evans Blue. However, this is a histological technique and is strongly influenced by adipocyte area, becoming inappropriate after HF diet-induced adipocyte hypertrophy^30, 32^. Using DCE-MRI, we demonstrate reduced pEAT blood flow in MG-supplemented groups and diabetic rats.

Several authors suggested that hypoxia could be caused by limited oxygen diffusion due to adipocyte hypertrophy, being a trigger to adipose tissue metabolic and endocrine dysfunction^4, 11, 12, 38, 39^. Nonetheless, the adipose tissue from obese patients was shown to be hyperoxic and to have only a very small proportion of adipocytes with a diameter superior to 100 µm, the oxygen diffusion distance, questioning such hypothesis^14, 40^. Our results demonstrate that HF diet-induced adipose tissue expansion does not cause significant alterations in adipose tissue blood flow and formation of hypoxic regions. Remarkably, hypoxia was found when the HF diet was combined with MG, showing that glycation-induced decreased blood flow leads to hypoxia when the adipose tissue is critically forced to expand.

Regarding the mechanisms involved in glycation-induced vascular lesions, some groups have introduced the concept of targeting adipose tissue angiogenesis to improve insulin sensitivity, based on decreased angiogenic ability of explants from obese donors^5, 15–18^. This is in line with observations showing that adipose tissue blood flow was observed to be decreased in obese patients due to impaired arteriolar function^41, 42^. Given that hypoxia is the main trigger for angiogenesis, we evaluated the role of glycation in regulating the mechanisms involved in adaptations to hypoxia and angiogenesis, as well as vascular tone. MG was shown to hamper HIF-1alpha stabilization in hypoxia, impairing cell response to hypoxia^43^. Here we show no differences in HIF-1alpha levels. Nevertheless, recent studies demonstrated the involvement of HIF-2alpha in preventing hypoxia-induced insulin resistance. Choe *et al*.^44^, demonstrated that HIF-2alpha expression in macrophages prevents M1 phenotype and their proinflammatory activity in adipose tissue. Mice lacking HIF-2alpha had insulin resistance and glucose intolerance^44^. Thus, while HIF-1alpha is important for the proinflammatory activation of M1 macrophages through iNOS induction, HIF-2alpha contributes to metabolic homeostasis by inhibiting such mechanisms^45^. Here we show decreased HIF-2alpha expression in high-fat diet-fed rats with MG supplementation and diabetic rats. Moreover, we observed an increased number of M1 macrophages in pEAT, which is in accordance with decreased HIF-2alpha levels. Such events may create a pro-inflammatory environment in the adipose tissue.

Our group recently demonstrated that MG-induced imbalance of VEGF/Ang-2 ratio inhibits tube-like formation, conducting to dysregulated endothelial cell proliferation and formation of aberrant capillaries^33^. In the present study, we show similar VEGF/Ang-2 ratio in HF diet-fed and control rats, but a decreased VEGF/Ang-2 ratio in HF diet-fed rats submitted to MG supplementation. Moreover, increased levels of the endothelial cell marker CD31 were observed, suggesting a higher number of endothelial cells and vessel disarrangement. Our data are also consistent with the findings of Jörgens *et al*.^46^, and Wang *et al*.^47^, which observed VEGF downregulation and formation of aberrant vessels in methylglyoxal-treated zebrafish and inhibition of angiogenesis in MG-treated human umbilical vein endothelial cells (HUVEC). The adipose tissue angiogenesis assay, an adaptation of the aortic ring assay, was recently developed by Corvera’s laboratory, but only for human subcutaneous and mice periepididymal adipose tissues^48, 49^. Based on the original one, we developed an assay for rat periepididymal adipose tissue. Our results show that, higher MG concentrations or selective inhibition of its detoxification by GLO-1 block the angiogenic process. However, before inhibiting cell proliferation and migration, MG destabilizes sprouts structure and affects their growth. This is in accordance with the findings of Liu *et al*.^50^, and Jörgens *et al*.^46^, who have shown excessive endothelial cell proliferation in HUVECs and formation of aberrant vessels in zebrafish after MG exposure. However, the mechanisms involved are still controversial. While the authors of the first study have shown increased autophagy-dependent VEGFR2 degradation, the others have shown increased VEGFR2 autophosphorylation, which would lead to excessive endothelial cell proliferation^46, 50^. Thus, MG has been shown to impair capillary structure and growth and here we extend these observations to adipose tissue vessels. Such events are likely to decrease adipose tissue blood flow and contribute to insulin resistance, but the mechanisms should be further elucidated in the future.

Karpe *et al*.^42^, observed impaired postprandial blood flow in adipose tissue and demonstrated that such events were associated with lower insulin sensitivity. As well, Farb *et al*., described impaired arteriolar function in the adipose tissue of obese patients, which mechanisms remain to be uncovered. Vascular tone is strongly influenced by the renin-angiotensin system (RAS) and by factors influencing vascular integrity and permeability. RAS has been shown to be upregulated by AGEs, increasing vascular damage in different tissues (reviewed by Matafome^9^). As well, AGEs were recently shown to increase vascular permeability by upregulating angiopoietin-like 4 (ANGPTL4) expression. Our results are in accordance with such body of evidence, showing that adipose tissue glycation increases AT1 and ANGPTL4 expression, which may impair vascular function.

Insulin resistance in fat depots contributes to increased spill-over of FFA to the circulation, ectopic deposition and consequently the development of all body insulin resistance^4, 51, 52^. Our present study demonstrates for the first time that accumulation of glycated products during pEAT expansion is associated with impaired insulin signalling in adipose tissue, Perilipin-A loss and decreased adiponectin secretion. Moreover, these alterations observed in pEAT contributed to systemic alterations, namely decreased glucose tolerance, hyperinsulinemia, increased FFAs and skeletal muscle insulin resistance. Our observations are in accordance with previous data from our laboratory and the studies of Gaens *et al*.^53, 54^, and Uribarri *et al*.^3^, demonstrating that RAGE-mediated CML accumulation in adipose tissue is involved in adipokines dysregulation and suggesting the involvement of AGE in the progression from healthy to unhealthy obesity.

**Figure 6:**
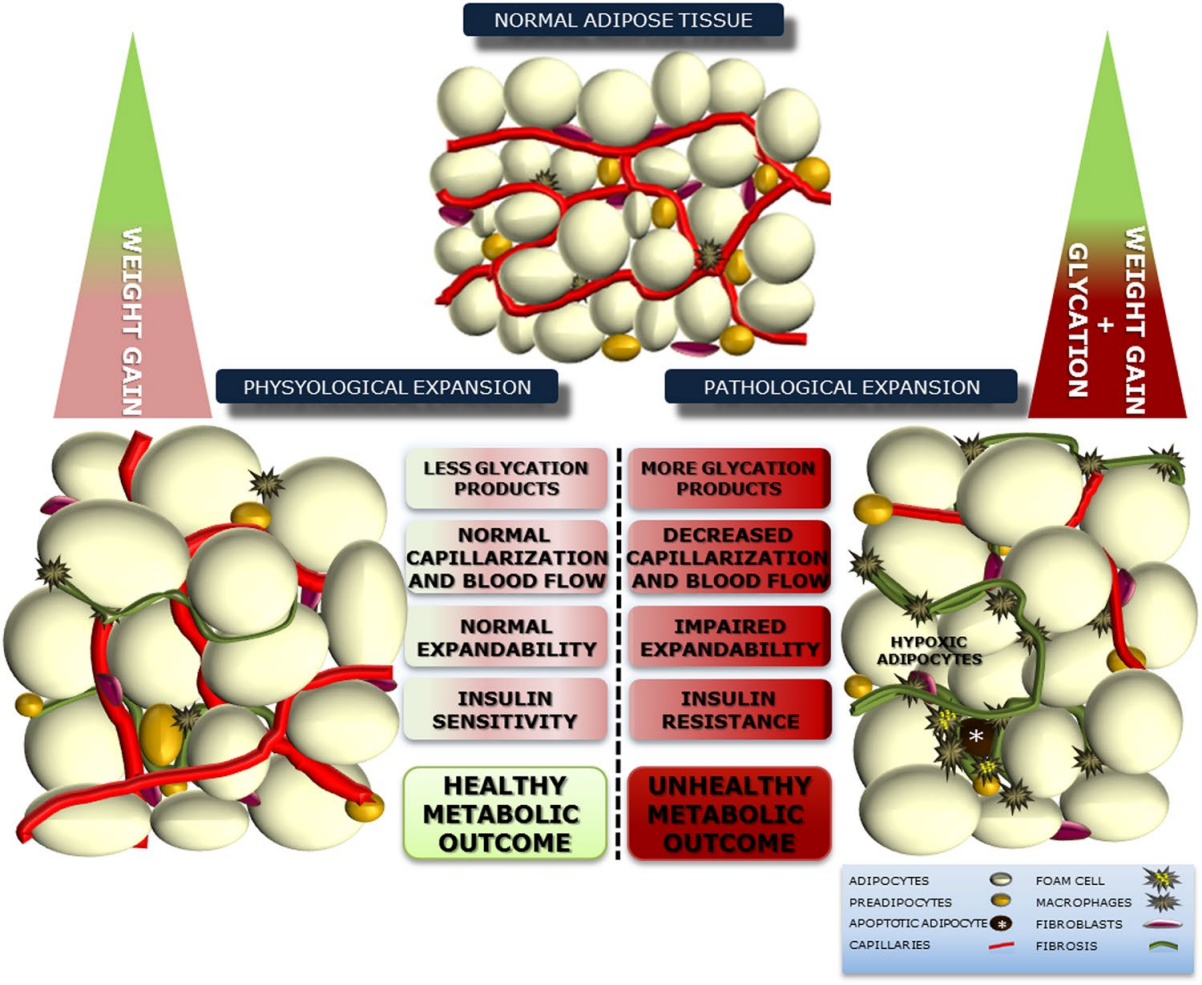
Impact of glycation in adipose tissue expandability, causing pathological expansion with impaired capillarization and blood flow, formation of hypoxc regions and insulin resistance. Such mechanisms may contribut to the poorer metabolic outcome of unhealthy obesity due to glucose dysmetabolism and alterations of the secretome.

In sum, our results demonstrate that glycation impairs pEAT microcirculation and expandability, in particular when associated with high fat diets and ensuing obesity. Moreover, such observations were replicated in the subcutaneous adipose tissue, showing that this is not a localized effect in pEAT (Supplementary Figure). Thus, we propose the existence of an adipovascular coupling mechanisms, based on the fact that blood flow is critical for adipocyte function and when decreased causes insulin resistance. This coupling is disrupted by glycation, impairing blood flow, adaptation to hypoxia and expansion potential, thus causing hypoxia and local and systemic insulin resistance (Fig. 6). Although the mechanisms should be further addressed in the future, our results suggest promising therapeutic targets in preventing unhealthy obesity and metabolic disorders.

## Research Design and Methods

### Antibodies

Calnexin, VEGFA, CD31, HIF-1 alpha, (AB0037, AB0063, AB0092, AB0112, Sicgen, Portugal), β-actin, α-tubulin (A5316, T6199, Sigma, USA), PPARγ, AKT, p-AKT(Ser473), VEGFR2, PI3K (#2443, #9272, #4058, #2479, #4249, Cell Signaling, USA), ANG-2, F4/80, Perilipin-A, GLUT4, p-IR(Y1361), GLO-I, C/EBPalpha, PGC1alpha, ANGPTL4, AT1, HIF-2 alpha (Ab8452, Ab74383, Ab3526, Ab65267, Ab60946, Ab96032, Ab40764, Ab191838, Ab2920, Ab9391, Ab8365, Abcam, UK), CD11c, CD206 (bs-2058R, bs-2664R Bioss, USA) TIE-2, IRβ (sc-324, sc-57342, SantaCruz Biotechnology, USA), CEL (KH025, TransGenic Inc, Japan).

### Animal maintenance

Wistar and Goto-Kakizaki (GK) rats from our breeding colonies (Faculty of Medicine, University of Coimbra) were kept under standard conditions^30, 32^. The experimental protocol was approved by the local Institutional Animal Care and Use Committee (ORBEA IBILI-FMUC 03-2015). All the procedures were performed in accordance with the European Union Directive for Protection of Vertebrates Used for Experimental and Other Scientific Ends (2010/63/EU) and by users licensed by the Federation of Laboratory Animal Science Associations (FELASA).

### Experimental groups

Male Wistar rats were randomly divided in four groups (n = 12/group): (1) Control (Ct) with standard diet AO3 (5% triglycerides, 21% proteins, 45% carbohydrates, SAFE, France); (2) Methylglyoxal group (MG) with standard diet and MG administration; (3) High-fat diet-fed group (HFD); (4) High-fat diet group with MG supplementation (HFDMG). A group (GK) of age-matched non-obese type 2 diabetic GK rats feeding the standard diet AO3 was used as a model of endogenous glycation.

### Diet and MG administration

High-fat (HF) diet (40% triglycerides, 10% carbohydrates and 26% proteins, 231 HF, SAFE, France) was administered during 18 weeks (8 to 12 months old). MG (75 mg Kg^−1^day^−1^) was administered orally as before^30, 32, 37^.

### Body weight and glycemic profile

In overnight (18 h) fasted rats, body weight was recorded and HbA1c, glycemia (fasting and 1 and 2 hours after i.p. glucose administration; 1.8 g Kg^−1^; IPGTT) were measured in the tail vein.

### Magnetic resonance imaging

A dynamic contrast-enhanced (DCE) MRI study was performed using a BioSpec 9.4 T MRI scanner (Bruke, Biospin, Ettlingen, Germany). Rats (n = 6/group) were kept anesthetized by isoflurane (2–3%) with 100% O_2_ with body temperature and respiration monitoring (Intruments SA, Stony Brook, USA). A body quadrature transmit/receive volume coil with 71/112 mm of inner/outer diameter was used. Images were acquired with a fat-saturated T1-weighted gradient-echo sequence with parameters: TR/TE = 301.5/2.5 ms, FA = 50°, FOV = 65 × 65 mm^2^, matrix size = 169 × 169, 40 slices (axial orientation), slice thickness = 1.0 mm, 40 dynamics, scan time per dynamic = 51 s, total scan time = 34 mins. The contrast agent (Gadovist®, LUSAL, Portugal) was administered intraperitoneally, after the acquisition of 3 baseline dynamics. Tissue enhancement curves were obtained offline using homemade software implemented in Matlab (v2013a, Mathworks, Natick, Mass). Intensity variation as a function of time was quantified in regions of interest (ROIs) in skeletal muscle, pEAT and subcutaneous adipose tissue. The area under the curve (AUC) was calculated to indirectly quantify blood flow.

### Blood and adipose tissue collection

Animals were anesthetized and serum and plasma were collected as described before^30, 32^. After sacrifice by cervical displacement, adipose and muscle tissue samples were frozen (−80 °C) or stored in 10% formalin.

### Analysis of adipose tissue hypoxic regions

Hypoxic regions in the adipose tissue were determined through intra-peritoneal injection of pimonidazole (60 mg^−1^ kg, 40 minutes, n = 3/group). The Hypoxia Probe Kit (Millipore, USA) was used to assess hypoxic regions by Western blotting (WB) and immunohistochemistry (IHC).

### Blood analyses

Serum triglyceride levels were determined using commercial kits (Olympus-Diagnóstica, Portugal). Plasma levels of FFA and insulin were assessed using the FFA Assay Kit (ZenBio, NC, USA) and the Rat Insulin ELISA Kit (Mercodia, Sweden). Serum adiponectin and leptin were determined using the Rat Adiponectin Immunoassay Kit and the Rat Leptin Immunoassay Kit (Invitrogen, USA).

### Western Blotting

Adipose tissue (300 mg) and skeletal muscle (100 mg) (n = 6) were homogenized and assayed as before^30, 32^. The secondary antibodies were anti-mouse (GE Healthcare, UK), anti-rabbit and anti-goat (Bio-Rad, USA). Membranes were revealed using ECL substrate in a Versadoc system (Bio-Rad, USA) and analyzed with Image Quant® (Molecular Dynamics, USA).

### Histological colorimetric assays

Tissue sections (4 µm) from paraffin-embedded pEAT (n = 3/group) were stained with Periodic Acid-Schiff (PAS) or Masson Trichrome staining. Images were captured in a Zeiss microscope with incorporated camera (Germany). The number of adipocytes was determined in at least 10 fields/slice and the mean adipocyte area was determined.

### Immunohistochemistry

Immune staining of pimonidazole adducts and CEL was performed after paraffin removal, hydration and blocking. Sections were incubated overnight (4 °C) with primary antibody and with secondary antibody-peroxidase (2 hours, RT) (IHC peroxidase Kit, Chemicon, USA). DAB (diaminobenzidine) was used as substrate. Sections were stained with hematoxylin before mounting.

### *Ex vivo* adipose tissue angiogenic assay

The rat adipose tissue angiogenic assay was developed based on the method described by Gealekman *et al*.^48^, and Rojas-Rodríguez *et al*.^49^. Periepididymal adipose tissue from 4 week old Wistar rats was collected and cut in ~1 mm^3^ pieces. Explants were then immediately embedded in 60 μl of collagen in 96-well plates and cultured with EGM-2 MV (BulletKit CC-3202; Lonza, Allendale, NJ, USA). Explants were incubated with control medium, MG-supplemented (50 μM, 100 μM, 250 μM, 50 μM and 1 mM), treated with the inhibitor of GLO-1 *S*-*p*-bromobenzylglutathione cyclopentyl diester (BBGC, 20 μM) or with BBGC 20 μM in combination with MG 250 μM (n = 15 explants/condition) (n = 4 experiments). After 6 days, images were captured in a Zeiss Axio Observer Z1 with an incorporated camera (Zeiss, Germany). The area of capillarization was calculated and normalized for the area of the explant. Tubular (sprout) length was also determined as a measure of cell organization and capillary integrity, as described before^55^.

### Statistical analysis

Results are presented as mean ± SEM per group. Given the relatively small sample size (n = 6–12), the non-parametric Kruskal-Wallis test (all pairwise multiple comparisons) was applied to determine all statistical differences between the groups, using the SPSS software (IBM, NY, USA). The alpha level of significance for all experiments was 0.05 and p < 0.05 was considered as the criterion for significance.

## Electronic supplementary material

Supplementary Information
