# Supplementary material for: Methylglyoxal-induced glycation changes adipose tissue vascular architecture, flow and expansion, leading to insulin resistance
Source: Sci Rep. 2017 May 10;7:1698. doi: 10.1038/s41598-017-01730-3 (PMC5431896; doi:10.1038/s41598-017-01730-3)
Supplement: Supplementary file 1 — Supplementary Information [file 41598_2017_1730_MOESM1_ESM.pdf]

Supplementary data

Methylglyoxal-induced glycation changes adipose tissue vascular architecture, flow and expansion, leading to insulin resistance

Tiago Rodrigues[1], Paulo Matafome[1,2*], José Sereno[3], José Almeida[1], João Castelhano[3], Luís Gamas[1], Christian Neves[1], Sónia Gonçalves[3], Catarina Carvalho[1], Amina Arslanagic[1], Elinor Wilcken[1], Rita Fonseca[1], Ilda Simões[4], Silvia Vilares Conde[5], Miguel Castelo-Branco[3,6], Raquel Seica[1]

1. Laboratory of Physiology, Institute of Biomedical Imaging and Life Sciences (IBILI), Faculty of Medicine, University of Coimbra, Portugal.
2. Instituto Politécnico de Coimbra, Coimbra Health School (ESTeSC), Department of Complementary Sciences, Coimbra, Portugal.
3. Institute of Nuclear Sciences Applied to Health (ICNAS), University of Coimbra.
4. Serviço de Anatomia Patológica, University Hospital Center of Coimbra.
5. CEDOC, NOVA Medical School, Faculty of Medical Sciences, New University of Lisbon
6. Laboratory of Visual Neuroscience, IBILI, Faculty of Medicine, University of Coimbra.

Supplementary figure 1 – Subcutaneous adipose tissue

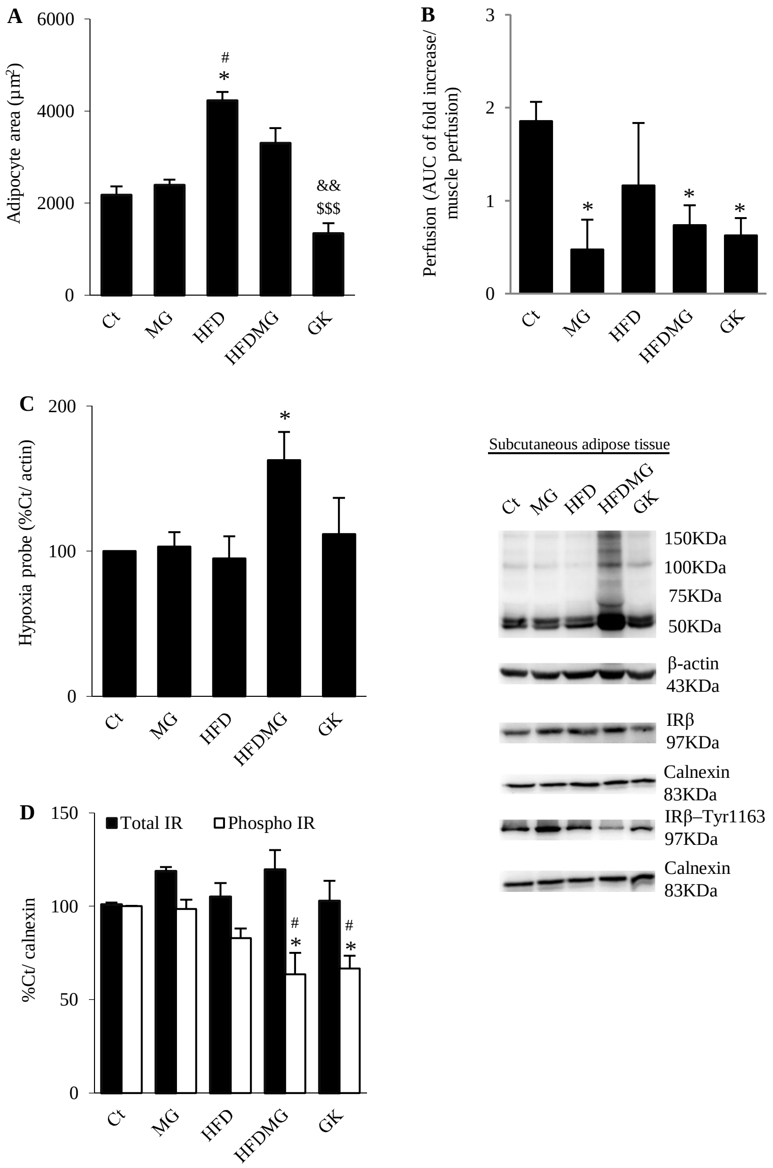

Supplementary figure 1: Methylglyoxal decreased subcutaneous adipose tissue blood flow and caused hypoxia and insulin resistance independently of adipocyte hypertrophy. Adipocyte area was evaluated through the number of adipocytes counted in each field (10 fields/rat) (A). Subcutaneous adipose tissue blood flow was evaluated through contrast product (gadolinium) accumulation using DCE-MRI (B). Hypoxia was assessed using an antibody against pimonidazole adducts by western blotting quantification (C). Insulin signaling was evaluated through western blotting quantification of activated and total forms of IR (D), as shown in representative images. Ct - Wistar 12 m; MG - Wistar + MG supplementation; HFD - HF diet-fed Wistar; HFDMG - HF diet-fed Wistar + MG supplementation; GK - Goto-Kakizaki 12m. Bars represent means ± SEM. * vs Ct; # vs MG; $ vs HFD; & vs HFDMG; 1 symbol $p<0.05$; 2 symbols $p<0.01$; 3 symbols $p<0.001$.

Supplementary figure 2 – MRI data validation

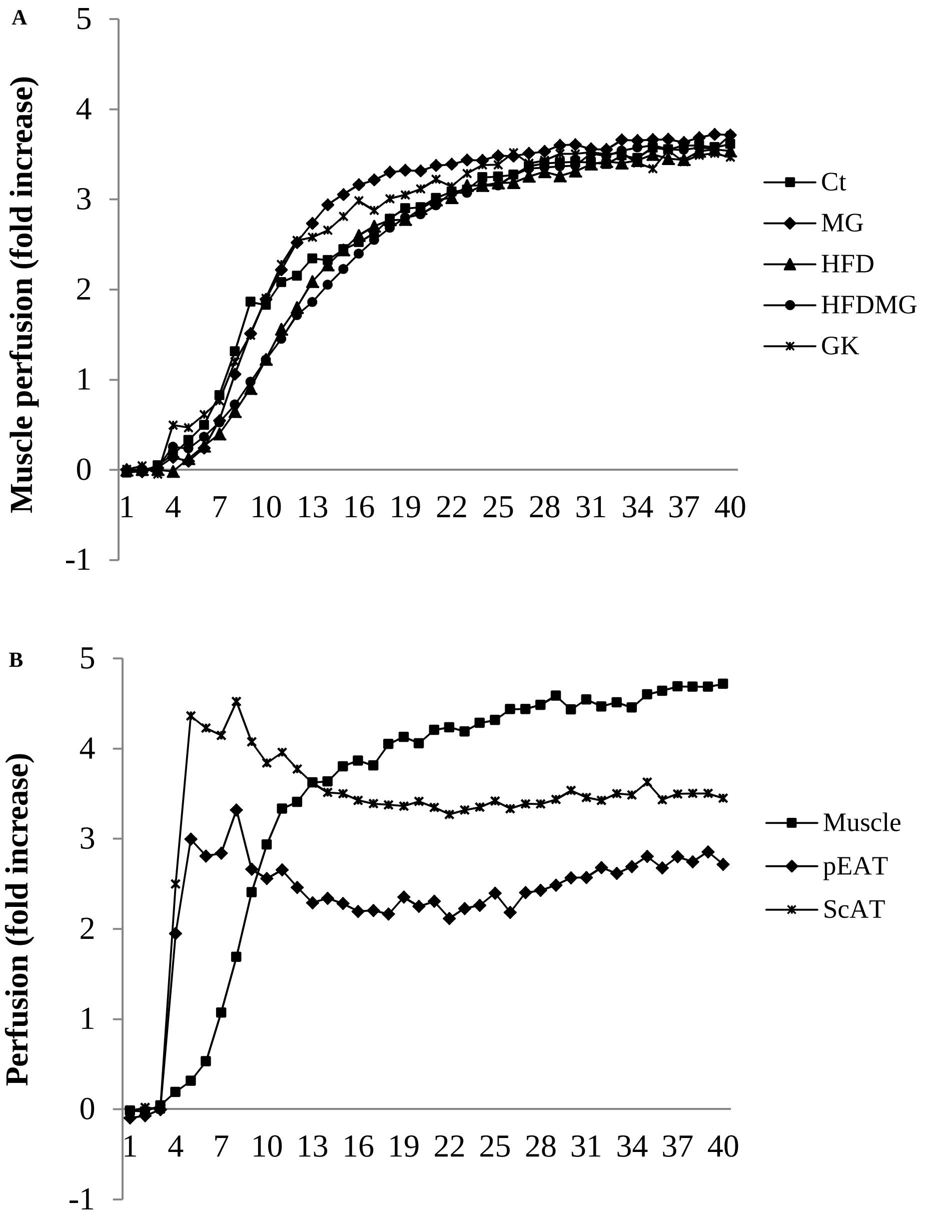

Supplementary figure 2: Graphic A shows mean values for each group at each dynamic during data acquisition (30 minutes), showing no significant differences in muscle perfusion between experimental groups.

Graphic B shows progressive accumulation of the contrast product in the skeletal muscle and periepididymal and subcutaneous adipose tissue of 6 months old Wistar rats, which were used as the control of the experiment. The representative curves show mean values for 4 rats. Besides the analysis of adipose tissue, we performed the additional control analysis of the perfusion data of the muscle. Interestingly, data show fast accumulation of the contrast product in adipose tissue and a more progressive accumulation in the muscle, followed by a period steady-state accumulation of the contrast product.
